# COVID-19 Vaccination Preferences of University Students and Staff in Hong Kong

**DOI:** 10.1001/jamanetworkopen.2022.12681

**Published:** 2022-05-17

**Authors:** Lydia W. Y. Fung, Jiaxi Zhao, Vincent K. C. Yan, Joseph E. Blais, Jacky C. H. Chan, Silvia T. H. Li, Jessica J. P. Shami, Christine Kwan, Yue Wei, Carlos K. H. Wong, Xue Li, Celine S. L. Chui, Eric Y. F. Wan, Francisco T. T. Lai, Samson Tse, Benjamin J. Cowling, Ian C. K. Wong, Esther W. Chan

**Affiliations:** 1 Centre for Safe Medication Practice and Research, Department of Pharmacology and Pharmacy, Li Ka Shing Faculty of Medicine, The University of Hong Kong, Hong Kong SAR, China; 2 Laboratory of Data Discovery for Health, Hong Kong Science and Technology Park, Hong Kong SAR, China; 3 Digital and Data Innovation, AstraZeneca Global R&D (China) Co Ltd, Shanghai, China; 4 Sau Po Centre on Ageing, The University of Hong Kong, Hong Kong SAR, China; 5 Department of Family Medicine and Primary Care, School of Clinical Medicine, Li Ka Shing Faculty of Medicine, The University of Hong Kong, Hong Kong SAR, China; 6 Department of Medicine, School of Clinical Medicine, Li Ka Shing Faculty of Medicine, The University of Hong Kong, Hong Kong SAR, China; 7 School of Nursing, Li Ka Shing Faculty of Medicine, The University of Hong Kong, Hong Kong SAR, China; 8 School of Public Health, Li Ka Shing Faculty of Medicine, The University of Hong Kong, Hong Kong SAR, China; 9 Department of Social Work and Social Administration, The University of Hong Kong, Hong Kong SAR, China; 10 Research Department of Practice and Policy, University College London, London, United Kingdom

## Abstract

**Importance:**

COVID-19 has required universities to rapidly develop vaccination policies for students and staff, yet little is known about the preferences of these individuals toward vaccination.

**Objective:**

To quantify student and staff preferences for COVID-19 vaccination at a university in Hong Kong.

**Design, Setting, and Participants:**

A cross-sectional online survey study was conducted from July 20 to September 21, 2021, before the announcement of a campus-wide vaccine mandate. A survey of 42 451 eligible university students and staff used discrete-choice experiment methods to quantify 7 attributes of COVID-19 vaccination: risk of a mild or moderate adverse event after vaccination, risk of a severe adverse event after vaccination, efficacy against COVID-19 infection, efficacy against severe manifestation of COVID-19 infection, duration of protection after vaccination, incentive for completing vaccination, and out-of-pocket costs.

**Main Outcomes and Measures:**

A mixed logit regression model was used to estimate the preferences of attributes for COVID-19 vaccines and marginal willingness to pay (mWTP) adjusted for background characteristics, role, vaccination, and COVID-19 infection status of family or friends, adverse event status after vaccination among family and friends of participants, and scenario block.

**Results:**

Among 42 451 eligible university students and staff invited, 3423 individuals completed the survey (mean [SD] age, 27.1 [9.9] years; 2053 [60.0%] women). Participants included 2506 students (73.2%) and 917 staff (26.8%), with a response rate of 8.1%. Quarantine-free travel was preferred (β = 0.86; 95% CI, 0.72-0.99; mWTP: $235.9; 95% CI, $190.3-$294.2), followed by efficacy against any COVID-19 infection (β = 0.30; 95% CI, 0.29-0.32; mWTP: $84.1; 95% CI, $71.8-$100.8), against severe manifestation of COVID-19 infection (β = 0.25; 95% CI, 0.24-0.27; mWTP: $69.7; 95% CI, $465-$653), and risk of severe adverse events following vaccination (β = −0.24; 95% CI, −0.27 to −0.21; mWTP: −$66.8; 95% CI, −$81.5 to −$55.3). Participants were less concerned about protection duration (β = 0.17; 95% CI, 0.15-0.18; mWTP: $46.0; 95% CI, $38.6-$56.2) and risk of mild to moderate adverse events (β = −0.12; 95% CI, −0.13 to −0.10; mWTP: −$32.7; 95% CI, −$41.2 to −$26.4).

**Conclusions and Relevance:**

Preference of all attributes were significant and were considered important by the participants for vaccine decision-making. Insights drawn could assist policy makers in future vaccination decisions, such as campus vaccine mandate and requirement of a third dose.

## Key Points

**Question:**

What are the preferences of university students and staff regarding COVID-19 vaccination in Hong Kong?

**Findings:**

In this survey study that included 3423 university students and staff, 7 attributes considering vaccine efficacy and safety, incentive for vaccination, and cost were assessed. Preferences on all attributes were found to be significant, with quarantine-free travel and vaccine efficacy against COVID-19 infection the most preferred factors; participants were less concerned about duration of protection and risk of potential mild to moderate adverse events.

**Meaning:**

The results of this study could support and promote COVID-19 vaccination through a better understanding of the perceived barriers and preferences among university students and staff.

## Teaser

This survey study examines the preferences of staff and students at the University of Hong Kong regarding receipt of the COVID-19 vaccine.

## Introduction

Since early 2020, the COVID-19 pandemic has severely disrupted teaching and learning activities at universities around the world, with substantial physical and mental health consequences to the members of these communities.^1,2,3^ In light of increasing COVID-19 vaccination rates and improving public health situations, many universities have resumed on-campus teaching since the 2021 autumn semester. Meanwhile, to ensure the safety and sustainability of campus activities, some universities or individual faculties have introduced a vaccine mandate requiring all staff and students to be fully vaccinated against COVID-19 or to undergo a weekly self-paid COVID-19 antigen test if they could not be vaccinated for medical or personal reasons.^4^ In May 2021, the university administration at The University of Hong Kong announced the resumption of on-campus teaching and learning activities and all current members of residential halls were required to be vaccinated or undergo weekly COVID-19 antigen tests on a self-paid basis^5,6^ (eTable 1 in the Supplement).

Enforcement of a vaccine mandate at universities has been a matter of debate worldwide. In the US, Rutgers University was the first university to mandate COVID-19 vaccination for students in March 2021,^7^ and other major universities have since followed.^8^ The recent tightening of vaccination and COVID-19 testing requirements for businesses in the US has further encouraged the enforcement of vaccine mandate at universities.^9^ In the UK, despite some universities introducing a vaccine mandate, the government has not implemented a mandatory requirement that university students be vaccinated for COVID-19.^10^ Aside from several universities in Hong Kong, a mandatory vaccination policy has not been implemented for most universities in Asia.^11,12^

Vaccination against COVID-19 is not a simple binary decision. Different populations prioritize different values and attributes in their decision-making, leading to a wide range of vaccine preferences.^13^ In Hong Kong, studies have assessed vaccine intention and vaccine hesitancy among the general public before the launch of the local vaccination campaign.^14,15,16,17^ Respondents in these studies reported low intention to receive COVID-19 vaccination. However, attributes influencing vaccination preferences among university students and staff in Hong Kong remain unclear, particularly as perceptions and preferences might have changed after the launch of the vaccination campaign when the vaccination policy was implemented and as more real-world evidence on COVID-19 vaccines became available.

Freedom of choice and public health priority have been a matter of debate alongside policies that have mandated compulsory vaccination. A better understanding of the decision-making process among students and staff is important to inform future vaccination and infection control policies for academic institutions. Therefore, this study used a survey with a discrete-choice experiment method to ascertain the preferences of university students and staff on vaccination against COVID-19.

## Methods

A cross-sectional internet-based survey with a discrete-choice experiment was conducted via Qualtrics (Qualtrics LLC) from July 20 to September 21, 2021, before the announcement of a campus-wide vaccine mandate (eTable 1 in the Supplement). We invited all current students and staff of The University of Hong Kong to participate in the study via the university bulk email system. This study was registered with ClinicalTrials.gov (NCT05150769). This study followed the American Association for Public Opinion Research (AAPOR) reporting guideline and was approved by the institutional review board of The University of Hong Kong/Hospital Authority of Hong Kong West Cluster. Informed consent was provided by respondents online before taking the survey. Participation was voluntary and all data were self-reported; participants were able to enter a drawing at completion of the survey for an electronic device to support research and education.

### Attribute and Level Identification

A total of 7 attributes of vaccination preferences were identified based on previous studies: risk of a mild or moderate adverse event after vaccination, risk of a severe adverse event after vaccination, efficacy against COVID-19 infection, efficacy against severe manifestation of COVID-19 infection, duration of protection after vaccination, incentive for completing vaccination, and out-of-pocket costs,^14,18,19,20,21^ in addition to review by an expert panel (eTable 2 in the Supplement). Four levels were used for all attributes. The levels of attributes that could reflect the safety and effectiveness profiles of COVID-19 vaccines, such as the risk of mild to moderate adverse events (redness, pain, swelling, fever, transient or short-term discomfort), the risk of severe adverse events (requiring extensive management/hospitalization and potential death), the efficacy for protection against COVID-19 infection, and the efficacy for protection against severe manifestation of COVID-19 infection were defined based on the mean value and 95% CI of those factors identified in pivotal randomized clinical trials. The levels of duration of protection and out-of-pocket costs were based on the literature and local costs of other vaccines. To obtain a better estimate of a linear association, all levels except for incentive upon completion of vaccination were set approximately evenly within each range.

### Discrete-Choice Experiment Design

A total of 24 scenarios with different combinations of attributes and levels were randomly selected from a generated pool of 4 × 4 × 4 × 4 × 4 × 4 × 4 = 16 384 combinations with orthogonality for each non–opt-out alternative.^22,23,24^ These 24 scenarios were evenly and randomly divided into 3 blocks of 8 scenarios and each participant was randomly allocated to 1 of 3 blocks (eTable 3 in the Supplement) in each questionnaire. To obtain accurate participant preferences for the provided attributes and avoid subjective association with specific vaccine brands, 2 unlabeled hypothetical vaccines were given in the scenarios for participants’ selection. The opt-out option (ie, regular antigen screening test) would be chosen if participants disliked both hypothetical vaccine options based on the attribute levels. In each scenario, an opt-out option was included to resemble real-life preference and maximize external validity.^25^

### Target Population and Sample Size

The questionnaire was sent to current students and staff at The University of Hong Kong, and responses from only those aged 18 years or older were kept for analyses. The minimum sample size was calculated according to the Orme rule of thumb as follows^26^: sample size greater than or equal to 500 × (largest level of attributes) / ([number of scenarios] × [number of non–opt-out alternatives]).

Given the conditions of 2 non–opt-out alternatives with a maximum of 4 attribute levels and 8 scenarios presented to each participant in our study design, at least 125 respondents were required. We aimed to recruit at least 300 participants to ensure the sample size had sufficient power for analysis.^27^

### Statistical Analysis

Mixed logit regression models were used to explore participants’ preferences on each attribute. Because the selection of opt-out option would be dependent on the selection of the other 2 options, scenarios with opt-out selected were removed from the main analysis to meet the assumption of independence of outcomes in mixed logit regression. The attribute for incentives upon completion of vaccination was considered a categorical variable, and the rest of the attributes were considered continuous variables (eTable 2 in the Supplement). Model coefficients (β) and 95% CIs were estimated.

Background characteristics and blocks of scenarios were adjusted in statistical analysis. Primary role (student/staff) was adjusted because these factors could influence participants’ understanding of the questionnaire. Factors potentially related to participants’ experience with COVID-19, such as the COVID-19 vaccination or infection status, and any adverse events following COVID-19 vaccination among family members and friends, were also adjusted.

Random effects were used for each attribute except out-of-pocket cost to control for unobserved heterogeneity due to residual confounding. Fixed effects were applied for out-of-pocket cost for full vaccination. The correlation between any pair of attributes was also adjusted in the model. Model goodness of fit was evaluated using the McFadden pseudo *R*^2^.

Marginal willingness to pay (mWTP) in US dollars (1 USD = 7.8 HKD) was calculated by the ratio of the coefficient of other attributes to the coefficient of out-of-pocket cost for full vaccination, which represented the cost that participants were willing to pay for an additional unit change in the attribute. Prevention of infection is the main objective for receiving the COVID-19 vaccine. Therefore, the ratios between the preference on efficacy for protection against COVID-19 infection and preferences on other attributes, including the risk of mild to moderate adverse events after vaccination, the risk of severe adverse events after vaccination, the efficacy for protection against severe manifestations of COVID-19 infection, and the duration of protection after full vaccination, were calculated to estimate how much of the risk and benefit from these attributes participants would accept if there was a 10% higher chance of protection against COVID-19 infection (eTable 4 in the Supplement). The ratios between the preferences on different incentives and the preference on efficacy for protection against COVID-19 infection were calculated to assess the magnitude of increase in the marginal percentage of protection against COVID-19 infection required to elicit the same subjective perception on receiving different incentives on the completion of vaccination. Subgroup analyses by sex, primary role (student/staff), current residential hall membership (yes/no), with or without mental health disorders (yes/no), and place of origin (local/nonlocal) were performed (eTable 5 in the Supplement).

Patient characteristics are summarized as mean (SD) for continuous variables and frequencies (percentage) for categorial variables. All hypothesis tests were 2-sided, with a significance level of .05. Discrete-choice experiments were designed using Ngene, version 1.1.4 (ChoiceMetrics). All statistical analyses were conducted using R, version 4.0.5 (R Foundation for Statistical Analysis).

## Results

### Participant Characteristics

We invited 42 451 students and staff at the university and received a total of 5210 responses over the study period. After excluding incomplete responses (eFigure in the Supplement), a total of 3423 participants completed the survey including 2506 students (73.2%) and 917 staff (26.8%), with mean (SD) age, 27.1 (9.9) years. A total of 1370 participants were men (40.0%) and 2053 were women (60.0%) (Table 1). The overall response rate was 8.1%.

**Table 1.** Characteristics of Participants

Characteristic	No. (%)
Total participants	3423
Age, mean (SD), y	27.1 (9.9)
Sex	
Male	1370 (40.0)
Female	2053 (60.0)
Place of origin	
Hong Kong	2461 (71.9)
Other cities in mainland China	530 (15.5)
India	61 (1.8)
South Korea	41 (1.2)
US	34 (1.0)
Malaysia	32 (0.9)
Other	264 (7.7)
Faculty	
Medicine	1010 (29.5)
Science	379 (11.1)
Social sciences	355 (10.4)
Engineering	343 (10.0)
Arts	273 (8.0)
Business and economics	271 (7.9)
Education	267 (7.8)
Law	163 (4.8)
Architecture	94 (2.7)
Dentistry	86 (2.5)
Other	182 (5.3)
Student	2506 (73.2)
Staff	917 (26.8)
Administration	361 (39.4)
Research	285 (31.1)
Technical	91 (9.9)
Nonprofessoriate teaching	87 (9.5)
Professoriate	71 (7.7)
Other academic	16 (1.7)
Clinical	6 (0.7)
Self-reported health conditions	
Mental health disorder	154 (4.5)
Autoimmune disease	54 (1.6)
Heart disease	29 (0.8)
Tumor (both benign and malignant)	23 (0.7)
Rheumatoid arthritis	18 (0.5)
Inflammatory bowel disease	16 (0.5)
Kidney disease	11 (0.3)
Respiratory disease	10 (0.3)
COVID-19 vaccination and infection status of friends and family	
Family members have received the COVID-19 vaccine	2862 (83.6)
Family members or friends have contracted COVID-19	1062 (31.0)
Family members or friends have experienced a severe adverse effect from the COVID-19 vaccine	444 (13.0)
Participant vaccination status	
Not vaccinated	618 (18.1)
Vaccinated	2805 (81.9)
Completed 2 doses	2351 (82.9)
Received first dose, will take second dose on time	439 (15.7)
Received first dose, will not take the second dose	45 (1.6)
Importance of factors when considering COVID-19 vaccination, mean (SD)^a^	
Responsibility as a Hong Kong resident	2.90 (1.65)
Self-protection	4.15 (1.20)
Peer pressure to receive vaccination	1.62 (1.42)
Peer pressure not to receive vaccination	1.38 (1.37)
Number of daily new COVID-19 cases	2.60 (1.54)
Vaccine technology (eg, mRNA, inactivated virus)	3.35 (1.50)
Vaccination leave (time off from work)	1.75 (1.66)
Current member of residential halls	
Yes	453 (13.2)
No	2970 (86.8)

Abbreviation: mRNA, messenger RNA.

^a^ Assessed with a 5-point Likert scale (1, least important; 5, most important).

### Main Analysis

Positive coefficients reflected participants’ favor and negative coefficients reflected participants’ disfavor of the specific attribute, and the absolute coefficient magnitude representing the weighting of the attribute was considered. For continuous variables, the change in coefficient magnitude corresponded to the change for each 10-percentage point increase (from 10% to 20%) or every 10-fold change (from 0.001% to 0.01%).

The preferences of all attributes were significant. Participants preferred quarantine-free travel (β = 0.86; 95% CI, 0.72-0.99), followed by efficacy for protection against COVID-19 infection (β = 0.30; 95% CI, 0.29-0.32), efficacy for protection against severe manifestation of COVID-19 infection (β = 0.25; 95% CI, 0.24-0.27), high-value incentives (β = 0.25), and risk of a potential severe adverse event after vaccination (β = −0.24; 95% CI, −0.27 to −0.21) (Table 2). Low-value incentives (β = 0.19) had a similar weight as the duration of protection (for every 3-month increase in protection, β = 0.17; 95% CI, 0.15-0.18). The lowest weight was the risk of a potential mild to moderate adverse event (for every 10% change, β = −0.12; 95% CI, −0.13 to −0.10; mWTP: −$32.7; 95% CI, −$41.2 to −$26.4).

**Table 2.** Overall Preference and Marginal Willingness to Pay of 3334 Participants in the Main Analysis

Attribute	Crude, β (95% CI)^a^	Adjusted, β (95% CI)^a^^,^^b^	mWTP^c^ (95% CI)
Cost	−0.00036 (−0.00044 to −0.00028)	−0.00047 (−0.00054 to −0.00039)	NA
Mild to moderate adverse events	−0.098 (−0.12 to −0.081)	−0.12 (−0.13 to −0.10)	−32.7 (−41.2 to −26.4)
Severe adverse events	−0.20 (−0.23 to −0.17)	−0.24 (−0.27 to −0.21)	−66.8 (−81.5 to −55.3)
Efficacy against COVID-19 infection	0.25 (0.23 to 0.27)	0.30 (0.29 to 0.32)	84.1 (71.8 to 100.8)
Efficacy against severe manifestation from COVID-19 infection	0.21 (0.19 to 0.24)	0.25 (0.24 to 0.27)	69.7 (59.6 to 83.7)
Duration of protection	0.14 (0.13 to 0.15)	0.17 (0.15 to 0.18)	46 (38.6 to 56.2)
Incentives			
Low value	0.25 (0.18 to 0.31)	0.19 (0.11 to 0.27)	52.2 (31.5 to 76.0)
High value	0.46 (0.37 to 0.55)	0.25 (0.14 to 0.36)	68.8 (37.7 to 103.7)
Quarantine-free travel	0.86 (0.75 to 0.96)	0.86 (0.72 to 0.99)	235.9 (190.3 to 294.2)
Model specifications			
Log likelihood	−12 582	−12 394	NA
McFadden pseudo *R*^2^	0.2400	0.2514	NA

Abbreviation: mWTP, marginal willingness to pay; NA, not applicable.

^a^ All differences significant at *P* < .001.

^b^ Adjusted for age, sex, primary role (student/staff), vaccination status of family or friends, COVID-19 infection status of people near participants, adverse event status after vaccination of people near participants, and scenario block.

^c^ Reported in USD, 1 USD = 7.8 HKD.

Participants were willing to pay the most ($235.9; 95% CI, $190.3-$294.2) if they were allowed quarantine-free travel. For the effectiveness and safety of the vaccine, participants were willing to pay $84.1 (95% CI, $71.8-$100.8) to obtain a 10% higher chance of protection against COVID-19 infection, $69.7 (95% CI, $465-$653) to obtain a 10% higher chance of protection against severe manifestation of COVID-19 infection, $66.8 (95% CI, −$81.5 to −$55.3) to reduce a 10-fold risk of a potential severe adverse event, $46.0 (95% CI, $38.6-$56.2) to obtain 3 additional months of protection, and $255 to reduce a 10% risk of a mild to moderate adverse event (Table 2).

To obtain a 10% higher chance of protection against COVID-19 infection, participants were willing to take 25.7% marginal risk of a mild to moderate adverse event or 12.6 times higher chance of a severe adverse event after vaccination. The range of risk of a severe adverse event was 0.001% to 1%, which represents a very low absolute risk. Participants perceived that the incentive of quarantine-free travel was equivalent to an additional 28.1% chance of protection against COVID-19 infection. However, the perceived benefit of incentives was only equivalent to an additional 6.2% chance for the low-value and 8.2% chance for the high-value incentives of protection against COVID-19 infection (eTable 4 in the Supplement).

Sensitivity analysis was performed to test the robustness of results. Efficacy of protection against COVID-19 infection and against severe manifestation of COVID-19 infection were treated as categorical variables to specify the relationship of effects (eTable 6 and eTable 7 in the Supplement).

### Subgroup Analysis

Although the magnitude of preferences appeared to differ in the comparison between university students and staff, the relative importance was similar (Table 3). Staff were willing to pay approximately double on attributes, except for incentives, compared with students.

**Table 3.** Preference and mWTP of Participants Stratified by Primary Role

Attribute	Student (n = 2435)		Staff (n = 899)	
	Adjusted β (95% CI)^a^	mWTP (95% CI)^b^	Adjusted β (95% CI)^a^	mWTP (95% CI)^b^
Cost	−0.00050 (−0.00060 to −0.00041)	NA	−0.00033 (−0.00050 to −0.00017)	NA
Mild to moderate adverse events	−0.16 (−0.18 to −0.14)	−41.2 (−52.1 to −32.8)	−0.23 (−0.27 to −0.18)	−87.2 (−172.1 to −55.5)
Severe adverse events	−0.25 (−0.29 to −0.21)	−63.5 (−80.3 to −50.3)	−0.30 (−0.37 to −0.22)	−113.5 (−220 to −72.6)
Efficacy against COVID-19 infection	0.32 (0.29 to 0.34)	80.4 (67.3 to 98.6)	0.38 (0.33 to 0.42)	144.7 (96.9 to 280.5)
Efficacy against severe manifestation from COVID-19 infection	0.26 (0.24 to 0.28)	65.4 (54.9 to 79.9)	0.31 (0.27 to 0.35)	117.7 (79.2 to 227.3)
Duration of protection	0.15 (0.14 to 0.17)	39.4 (32.1 to 49.4)	0.17 (0.14 to 0.19)	64.2 (41.0 to 128.2)
Incentives				
Low value	0.25 (0.15 to 0.34)	62.4 (37.7 to 90.5)	0.39 (0.22 to 0.55)	148 (77.1 to 304.2)
High value	0.24 (0.11 to 0.37)	61.4 (27.8 to 99.9)	0.16 (−0.093 to 0.40)	59.7 (−36.5 to 187.4)
Quarantine-free travel	0.78 (0.63 to 0.93)	197.2 (149.9 to 257.2)	0.71 (0.44 to 0.98)	273 (150.9 to 562.3)
Model specifications				
Likelihood	−9059	NA	0.2516	NA
McFadden pseudo *R*^2^	−3230	NA	0.2740	NA

Abbreviation: mWTP, marginal willingness to pay; NA, not applicable.

^a^ Adjusted for age, sex, primary role (student/staff), vaccination status of family or friends, COVID-19 infection status of people near participants, adverse event status after vaccination of people near participants, and scenario block.

^b^ Reported in USD, 1 USD = 7.8 HKD.

The preferences of current members of residential halls were further explored (Table 4). The relative importance between other attributes and efficacy for protection against COVID-19 infection was similar to those in the main analyses. Willingness to pay for all attributes, except for incentives among current members, were less than those for nonmembers.

**Table 4.** Preference and Marginal Willingness to Pay of Participants Stratified by Current Membership in Residential Halls

Attributes	Current resident (n = 446)		Not a current resident (n = 2888)	
	Adjusted β (95% CI)^a^	mWTP (95% CI)^b^	Adjusted β (95% CI)^a^	mWTP (95% CI)^b^
Cost	−0.00079 (−0.0010 to −0.00054)	NA	−0.00042 (−0.00050 to −0.000033)	NA
Mild-to-moderate adverse events	−0.16 (−0.21 to −0.10)	−25.3 (−39.2 to −15.9)	−0.18 (−0.21 to −0.16)	−56.7 (−73.3 to −45.4)
Severe adverse events	−0.18 (−0.28 to −0.085)	−29.7 (−52.3 to −13.6)	−0.26 (−0.30 to −0.23)	−81.4 (−104.7 to −64.6)
Efficacy against COVID-19 infection	0.30 (0.24 to 0.36)	48.6 (35.6 to 71.2)	0.34 (0.32 to 0.37)	105.6 (87.3 to 132.6)
Efficacy against severe manifestation from COVID-19 infection	0.25 (0.21 to 0.30)	40.8 (30.5 to 58.5)	0.27 (0.25 to 0.29)	84.2 (69.5 to 105.6)
Duration of protection	0.14 (0.10 to 0.18)	22.7 (15 to 35.4)	0.16 (0.15 to 0.18)	49.5 (40 to 63.6)
Incentives				
Low value	0.21 (−0.019 to 0.44)	34.4 (−2.9 to 75.9)	0.32 (0.23 to 0.29)	98.3 (68.8 to 136.3)
High value	0.23 (−0.082 to 0.54)	36.7 (−13.8 to 94.6)	0.19 (0.064 to 0.32)	59.2 (20.5 to 103.5)
Quarantine-free travel	1.02 (0.67 to 1.39)	165.9 (165.9 to 265.1)	0.73 (0.59 to 0.88)	225.5 (170.1 to 299.9)
Model specifications				
Log likelihood	−1784	NA	−10 482	NA
McFadden pseudo *R*^2^	0.2308	NA	0.2637	NA

Abbreviation: mWTP, marginal willingness to pay.

^a^ Adjusted for age, sex, primary role (student/staff), vaccination status of family or friends, COVID-19 infection status of people near participants, adverse event status after vaccination of people near participants, and scenario block.

^b^ Reported in USD, 1 USD = 7.8 HKD.

Participants with mental health disorders were less willing to pay for most of the attributes compared with those without mental health disorders (Table 5). Participants with mental health disorders were less concerned about the risk of mild to moderate adverse events after vaccination, as suggested by the willingness to take a 32.8% increased risk in this attribute to obtain a 10% higher chance of protection against COVID-19 infection. The perceived benefit of quarantine-free travel for participants with mental health disorders was equivalent to an additional 44.8% chance of protection against COVID-19 infection, suggesting that quarantine-free travel is an important factor in the decision-making process for vaccination (eTable 4 in the Supplement). Vaccination preferences were similar when comparing local with nonlocal participants (eTable 5 in the Supplement).

**Table 5.** Preference and mWTP of Participants Stratified by Self-reported History of Mental Disorder

Attributes	Mental health disorder (n = 146)		No mental health disorder (n = 3188)	
	Adjusted β (95% CI)^a^	mWTP (95% CI)^b^	Adjusted β (95% CI)^a^	mWTP (95% CI)^b^
Cost	−0.0053 (−0.0089 to −0.0017)	NA	−0.00045 (−0.00053 to −0.00037)	NA
Mild-to-moderate adverse events	−0.51 (−0.81 to −0.22)	−12.6 (−30.8 to −6.4)	−0.18 (−0.20 to −0.16)	−50.8 (−63.1 to −41.4)
Severe adverse events	−1.54 (−2.69 to −0.39)	−37.4 (−66.8 to −16.8)	−0.25 (−0.29 to −0.22)	−72.6 (−91.3 to −58.5)
Efficacy against COVID-19 infection	1.69 (0.71 to 2.66)	41.0 (28.6 to 71.0)	0.32 (0.30 to 0.34)	92.2 (77.7 to 112.9)
Efficacy against severe manifestation from COVID-19 infection	1.82 (0.74 to 2.89)	44.2 (30.6 to 75.1)	0.26 (0.25 to 0.28)	75.3 (63.6 to 91.4)
Duration of protection	0.90 (0.31 to 1.49)	22.1 (11.3 to 43.7)	0.16 (0.14 to 0.17)	44.4 (36.7 to 55.4)
Incentives				
Low value	1.65 (−0.45 to 3.75)	40 (−19.0 to 12.7)	0.28 (0.19 to 0.36)	79.0 (53.7 to 109.1)
High value	3.73 (0.0096 to 7.45)	90.8 (−1.41 to 173.5)	0.22 (0.10 to 0.33)	61.7 (28.6 to 98.3)
Quarantine-free travel	7.54 (1.28 to 13.81)	183.6 (54.4 to 335.6)	0.75 (0.61 to 0.88)	213.2 (166.8 to 274.4)
Model specifications				
Log likelihood	−474	NA	0.3696	NA
McFadden pseudo *R*^2^	−11 806	NA	0.2530	NA

Abbreviation: mWTP, marginal willingness to pay.

^a^ Adjusted for age, sex, primary role (student/staff), vaccination status of family or friends, COVID-19 infection status of people near participants, adverse event status after vaccination of people near participants, and scenario block.

^b^ Reported in USD, 1 USD = 7.8 HKD.

## Discussion

To our knowledge, this is the first study in which vaccination preferences of both students and staff at a university community were examined, as previous studies mainly surveyed the general population or university students without WTP analysis.^17,28^ Compared with a representative population sample, university students and staff have experienced the suspension of face-to-face teaching and work for over a year, and this change may have considerably affected their mental, physical, and financial well-being or influenced their vaccination preference.^2^

The results showed that quarantine-free travel was an important consideration. International travel was customary among the university community before the pandemic. However, global travel restrictions implemented in the early stages of the pandemic led to the suspension of activities requiring travel, such as conference attendances, exchanges to foreign universities, and trips to their home countries for international students. Furthermore, there might be an association between urban density and the frequency of long-distance and leisure travel.^29^ Hong Kong being a highly urbanized city would have contributed to the preference for quarantine-free travel among the participants owing to the lack of recreational opportunities. The available evidence has reported that quarantine imposes a negative psychological impact,^30^ which could exacerbate mental health disorders, including depression and anxiety.^31^ This finding is consistent with our results, as participants with mental health disorders place greater weighting on quarantine-free travel when making a vaccine decision, compared with participants without mental health disorders.

Following quarantine-free travel, the efficacy against COVID-19 infection, protection against the severe manifestation of COVID-19 infection, and risk of potential severe adverse events following vaccination were also preferred. These findings suggested that participants were mostly concerned with the effectiveness of the vaccine per se when making a vaccine decision. The findings are consistent with a study on the factors of COVID-19 vaccine hesitancy in Hong Kong.^15^ As clinical trials and discussions about the COVID-19 vaccine booster continue,^32^ understanding of effectiveness as the dominant factor that influences respondent vaccine choice would facilitate vaccine uptake in future campaigns.

However, although the perceived concern over safety of the COVID-19 vaccine was a major factor contributing to vaccine hesitancy and a barrier to vaccine uptake, our findings suggest that people were generally willing to accept a certain level of risk of adverse events for the benefits of protection from COVID-19 infection and its severe consequences. Our study quantified the magnitude of this trade-off and found that people were willing to take a 25.7% additional risk of mild to moderate adverse events, and 12.6 times higher chance of severe adverse events, for an extra 10% chance of protection from COVID-19 infection The value of quarantine-free travel was perceived to be equivalent to a 28.1% higher chance of protection from COVID-19 infection. These findings suggest that the perceived fear of adverse events after vaccination is not insurmountable. The mean age of the study population was younger compared with the general population, which agrees with results from a study in China reporting that younger individuals were more favorably disposed to COVID-19 vaccination.^33^ Emphasis on the comparative benefit of protection from COVID-19 infection and tangible incentives, such as quarantine-free travel, could be useful strategies for promoting vaccination among university students and staff.

At the time of this study, only universities introduced vaccine mandate policies in Hong Kong. Because vaccine mandate policies may be perceived to constrain individual freedom of choice for the benefit of public health, the implementation of such policies was expected to raise concerns among the university’s stakeholders.

To promote vaccination awareness among students and staff and alleviate potential concerns and anxiety regarding vaccination, the university has introduced a variety of supportive measures, such as myth-breaking webinars on COVID-19 vaccination^34^ and 2 on-campus vaccination days.^35^ The university provided recommended tenders for COVID-19 test kits to ensure affordable antigen test kits for individuals who preferred regular self-paid tests and free test kits to students and staff who are medically verified as unsuitable for vaccination. Support was provided to students and staff with mental health disorders through promotion of mindfulness, counselling and psychological services. It is anticipated that these supportive measures will further smooth the implementation of the vaccine mandate and overcome perceived barriers toward COVID-19 vaccination.

In light of the issue of waning immunity of 2 vaccine doses and the resurgence of cases caused by new variants, administration of a booster (third) dose has been rapidly implemented for priority groups, such as older individuals and vulnerable populations, in different countries and regions. Study results showed that the additional dose would elicit a better immune response.^36^ The third dose for Hong Kong residents aged 12 years and older became mandatory on May 31, 2022.

### Limitations

This study has limitations. Similar to other preference surveys, behavioral intentions in this study were elucidated based on hypothetical scenarios and the true preferences of participants might not be adequately revealed. There could be other attributes not included in our questionnaire that would reflect additional preferences. Nevertheless, the 7 attributes included in the study were noted to be of most concern. Low overall response rate was also a limitation of the study, yet the number of complete responses exceeded the minimum sample size required by the Orme rule of thumb to generate sufficient statistical power for analysis. The distribution of respondents also showed a similar pattern with the distribution of students and staff from different faculties.^37,38^ Generalizability of our results to the general population may be limited; however, the results may be generalizable to other universities in Asia or universities with stringent vaccination policies, which was the aim of our study. The University of Hong Kong is the largest university in Hong Kong with the highest number of students and staff; therefore, the sample would be representative of the overall university populations in Hong Kong.^37,38,39^ At the time of the study, COVID-19 vaccines were provided free in Hong Kong. Although this ease of access might limit the interpretability of WTP results, we aimed to provide a quantitative angle of preferences by understanding respondents’ willingness to pay for a vaccine, which could inform similar health care policy decision-making in the future.^40^

## Conclusions

In this survey of Hong Kong university students and staff, the preferences for all attributes of COVID-19 vaccination examined in this study were significant. Quarantine-free travel and protection against COVID-19 infection and severe manifestation of infection were the primary considerations for receiving the COVID-19 vaccine among university students and staff. Insights drawn from this study could assist policy makers in future vaccination decisions, such as vaccine mandates for university students and staff, and the requirement of a third dose of COVID-19 vaccine.

## Notes

Supplement. **eTable 1.** Chronology of COVID-19-Related Teaching and Working Arrangements and Implementation of Policies at The University of Hong Kong (HKU), 2020-2021 **eFigure.** Flowchart of Participant Recruitment and Inclusion in the Discrete Choice Experiment at the University of Hong Kong **eTable 2.** Attributes and Levels Assessed During the Discrete Choice Experiment on COVID-19 Vaccination **eTable 3.** Example of Scenarios Presented to Participants **eTable 4.** Risk-Benefit Analysis of Attributes Among Different Subgroups **eTable 5.** Preference Weights and Marginal Willingness to Pay (mWTP) in USD of Participants Stratified by Place of Origin **eTable 6.** Preference Weights Estimated by Mixed Logit Regression Model With Efficacy of Protection Against COVID-19 Infection as a Categorical Variable **eTable 7.** Preference Weights Estimated by Mixed Logit Regression Model With Efficacy of Protection Against Severe Manifestation of COVID-19 Infection as a Categorical Variable
