# Supplementary material for: COVID-19 Vaccination Preferences of University Students and Staff in Hong Kong
Source: JAMA Netw Open. 2022 May 17;5(5):e2212681. doi: 10.1001/jamanetworkopen.2022.12681 (PMC9115609; doi:10.1001/jamanetworkopen.2022.12681)
Supplement: Supplement. — eTable 1. Chronology of COVID-19-Related Teaching and Working Arrangements and Implementation of Policies at The University of Hong Kong (HKU), 2020-2021 eFigure. Flowchart of Participant Recruitment and Inclusion in the Discrete Choice Experiment at the University of Hong Kong eTable 2. Attributes and Levels Assessed During the Discrete Choice Experiment on COVID-19 Vaccination eTable 3. Example of Scenarios Presented to Participants eTable 4. Risk-Benefit Analysis of Attributes Among Different Subgroups eTable 5. Preference Weights and Marginal Willingness to Pay (mWTP) in USD of Participants Stratified by Place of Origin eTable 6. Preference Weights Estimated by Mixed Logit Regression Model With Efficacy of Protection Against COVID-19 Infection as a Categorical Variable eTable 7. Preference Weights Estimated by Mixed Logit Regression Model With Efficacy of Protection Against Severe Manifestation of COVID-19 Infection as a Categorical Variable [file jamanetwopen-e2212681-s001.pdf]

# Supplementary Online Content

Fung LWY, Zhao J, Yan VKC, et al. COVID-19 vaccination preferences of university students and staff in Hong Kong. *JAMA Netw Open*. 2022;5(5):e2212681. doi:10.1001/jamanetworkopen.2022.12681

**eTable 1.** Chronology of COVID-19-Related Teaching and Working Arrangements and Implementation of Policies at The University of Hong Kong (HKU), 2020-2021

**eFigure.** Flowchart of Participant Recruitment and Inclusion in the Discrete Choice Experiment at the University of Hong Kong

**eTable 2.** Attributes and Levels Assessed During the Discrete Choice Experiment on COVID-19 Vaccination

**eTable 3.** Example of Scenarios Presented to Participants

**eTable 4.** Risk-Benefit Analysis of Attributes Among Different Subgroups

**eTable 5.** Preference Weights and Marginal Willingness to Pay (mWTP) in USD of Participants Stratified by Place of Origin

**eTable 6.** Preference Weights Estimated by Mixed Logit Regression Model With Efficacy of Protection Against COVID-19 Infection as a Categorical Variable

**eTable 7.** Preference Weights Estimated by Mixed Logit Regression Model With Efficacy of Protection Against Severe Manifestation of COVID-19 Infection as a Categorical Variable

This supplementary material has been provided by the authors to give readers additional information about their work.

© 2022 Fung LWY et al. *JAMA Network Open.*

**eTable 1**. Chronology of COVID-19-related teaching and working arrangements and implementation of policies at The University of Hong Kong (HKU), 2020-2021

| Date | Event |
|---|---|
| 29 January 2020 | Suspension of face-to-face teaching and commencement of online teaching for HKU students |
| 23 February 2020 | First COVID-19 cases confirmed in Hong Kong |
| March 2020 | HKU staff required to work from home with small numbers of staff rotating to provide basic departmental services |
| July 2020 | Continuation of online teaching for HKU students announced for the 2020/21 academic year |
| 26 February 2021 | Launch of community vaccination programme with Sinovac vaccines in Hong Kong |
| 6 March 2021 | Pfizer-BioNTech vaccines available at community vaccination centres in Hong Kong |
| 12 March 2021 | Launch of HKU Community Vaccination Centre in Ap Lei Chau for staff and students (open to public on 16 March 2021) |
| 5 May 2021 | Announcement on resuming face-to-face teaching commencing September 2021 |
| 21 May 2021 | Announcement of requirement for residents at student halls and colleges of The University of Hong Kong to be completely vaccinated against COVID-19 by September 2021 or take weekly self-paid antigen test |
| 20 July 2021 – 21 September 2021 | Study survey period |
| 11 November 2021 | Announcement on vaccine mandate for all students and staff at The University of Hong Kong effective from 17 January 2022 |
| 17 January 2022 | Campus-wide mandatory vaccination or antigen test to take effect (first day of Spring Semester, 2021/2022) |

© 2022 Fung LWY et al. *JAMA Network Open.*

**eFigure**. Flowchart of participant recruitment and inclusion in the discrete choice experiment at the University of Hong Kong

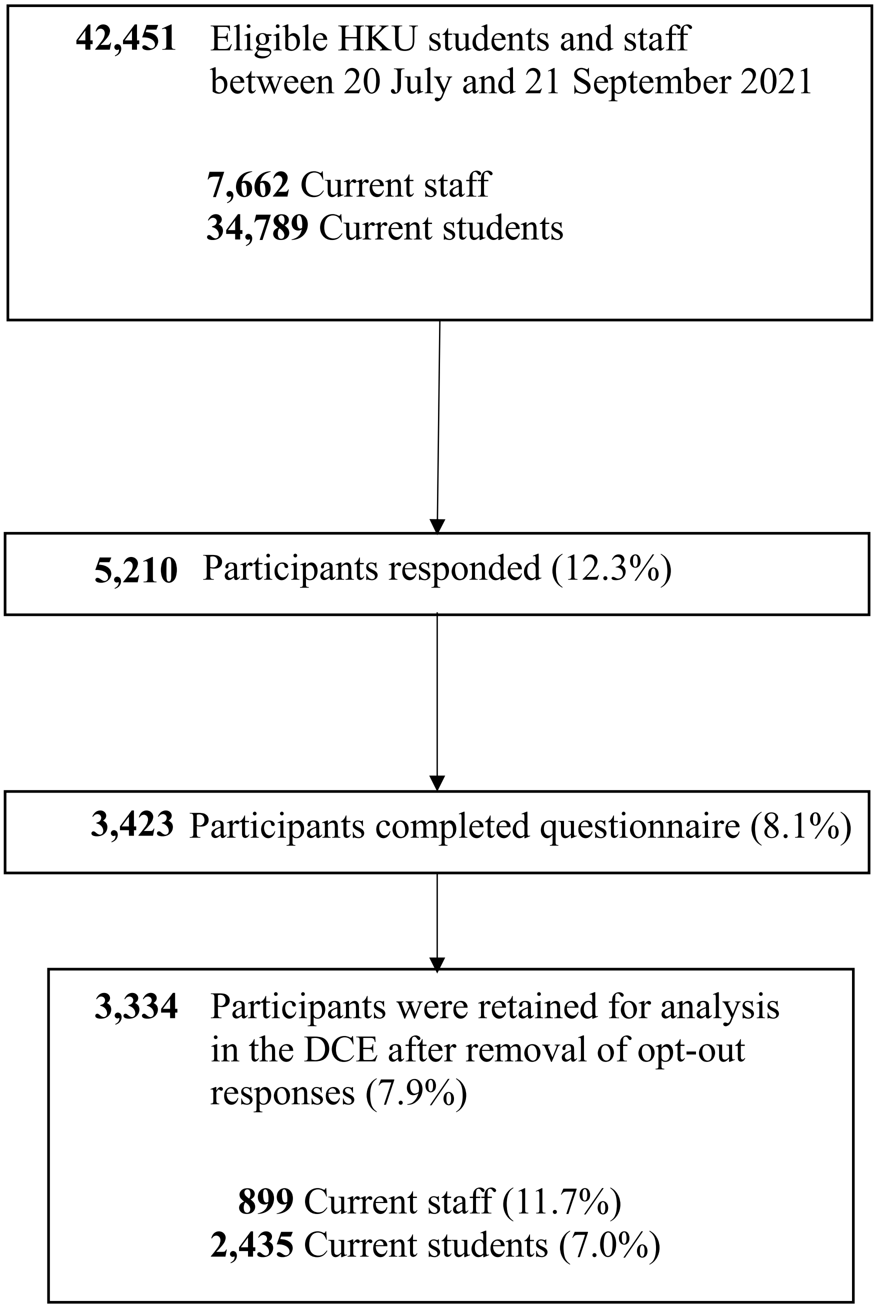


© 2022 Fung LWY et al. *JAMA Network Open.*

**eTable 2.** Attributes and levels assessed during the discrete choice experiment on COVID-19 vaccination

| **Attributes** | **Levels**[1] |
|---|---|
| Out-of-pocket cost for full vaccination (HKD) | 0/400/800/1000 |
| Risk of mild-moderate adverse events after vaccination (%)[2] | 10/30/50/70 |
| Risk of severe adverse events after vaccination (%)[3] | <0.001/<0.01/<0.1/<1 |
| Efficacy for protection against COVID-19 infection (%) | 30/50/70/95 |
| Efficacy for protection against severe manifestation of COVID-19 infection (%)[4] | 20/50/80/100 |
| Duration of protection after full vaccination (months) | 0/6/12/24 |
| Incentive upon completion of vaccination | None (reference) /Low value/High value/ Quarantine-free travel[5] |

[1] To allow for comparison of the scales of all attributes (except for out-of-pocket costs), the levels of risk of mild-to-moderate adverse events after vaccination were set to be 1/3/5/7 which represent 10%/30%/50%/70%, such that each unit change represents a 10% additional risk. The levels of risk of severe adverse event after vaccination were transformed from the logarithmic scale to linear scale, where 1/2/3/4 represents <0.001%/<0.01%/<0.1%/<1% respectively, such that each unit change represents a 10-fold risk. The levels of efficacy for protection against COVID-19 infection were set to be 3/5/7/9.5 which represent 30%/50%/70%/95%, such that each unit change represents 10% additional protection. The levels of efficacy for protection against severe manifestation of COVID-19 infection were set to be 2/5/8/10 which represents 20%/50%/80%/100%, such that each unit change represents 10% additional protection; and the levels of duration of protection after full vaccination (months) were set to be 0/2/4/8 which represents 0/6/12/24 month(s), where each unit change represents three-months of additional protection.

[2] Mild-to-moderate adverse events include, but not limited to, redness, pain, swelling, fever, transient or short-term discomfort.

[3] Severe adverse events include, but not limited to, those requiring extensive management or hospitalization, and potential death.

[4] Examples of severe manifestation of COVID-19 infection include requiring respiratory support or resuscitation and requiring intensive care unit admission.

[5] Low-value incentive refers to souvenirs; high-value incentive refers to prizes won from vaccinee-only lucky draw events.

© 2022 Fung LWY et al. *JAMA Network Open.*

**eTable 3.** Example of scenarios presented to participants

In this section, please consider the following 8 hypothetical scenarios, and choose ONE of the THREE options presented:

1. Vaccine A
2. Vaccine B
3. Antigen testing

Please note that:

- Examples of mild-to-moderate adverse events include (but not limited to): redness, pain, swelling, fever, transient or short-term discomfort.
- Examples of severe adverse events include (but not limited to): those requiring extensive management/hospitalization; potential death.
- Corresponding % is the chance of occurrence, and “N/A” stands for Not applicable.

| Scenario 1 | Vaccine A | Vaccine B | Antigen Test |
|---|---|---|---|
| Mild-to-moderate adverse events (%) | 30 | 50 | 0 |
| Severe adverse events (%) | <0.1 | <1 | 0 |
| Efficacy for protecting against COVID-19 infection (%) | 50 | 70 | 0 |
| Efficacy for protecting against severe manifestation of COVID-19 infection (%) | 50 | 80 | 0 |
| Duration of protection after completion of 2 vaccine doses (~months) | 24 | 3 | 0 |
| Incentive upon completion of vaccination | Souvenir (low value) | None | None |
| Out-of-pocket cost (HKD) | 400 | 800 | 240 (weekly) |

© 2022 Fung LWY et al. *JAMA Network Open.*

**eTable 4.** Risk-benefit analysis of attributes among different subgroups

| | Overall | Students | Staff | Current member of residential halls | Not current member of residential halls | With mental health disorder | Without mental health disorder |
|---|---|---|---|---|---|---|---|
| **To obtain a 10% more chance of protecting against COVID-19 infection** | | | | | | | |
| Marginal risk of encountering a mild-to-moderate adverse event after vaccination that participants would like to take (%) | 25.7 (22.7, 29.5) | 19.5 (17.3, 22.4) | 16.6 (14.2, 20.0) | 19.3 (14.2, 28.0) | 18.6 (16.8, 21.0) | 32.8 (17.6, 66.7) | 18.2 (16.4, 20.3) |
| Marginal risk of encountering a severe adverse event after vaccination that participants would like to take (times) | 12.6 (11.2, 14.4) | 12.7 (11.0, 14.9) | 12.7 (10.3, 16.5) | 16.3 (10.5, 34.0) | 13.0 (11.5, 15.0) | 10.9 (8.0, 21.0) | 12.7 (11.2, 14.7) |
| Marginal benefit of protection against severe manifestation of COVID-19 infection that participants would like to give up (%) | 12.0 (11.3, 12.9) | 12.3 (11.4, 13.3) | 12.3 (11.0, 13.7) | 11.9 (9.7, 14.6) | 12.5 (11.7, 13.4) | 9.3 (7.1, 12.4) | 12.3 (11.5, 13.0) |
| Marginal duration of protection of the vaccine that participants would like to give up (month) | 5.5 (5.1, 5.9) | 6.1 (5.6, 6.8) | 6.8 (5.8, 8.0) | 6.4 (5.0, 8.6) | 6.4 (5.9, 7.0) | 5.6 (4.0, 8.9) | 6.4 (5.7, 6.8) |
| **Perceived benefit of incentive expressed in terms of extra vaccine protection (%)** | | | | | | | |
| Low-value incentive | 6.2 (3.7, 8.7) | 7.8 (4.9, 10.8) | 10.2 (6.1, 14.5) | 7.1 (-0.7, 15.3) | 9.3 (6.8, 11.9) | 9.8 (-3.6, 23.4) | 8.6 (6.0, 11.0) |
| High-value incentive | 8.2 (4.5, 12.0) | 7.6 (3.4, 11.8) | 4.1 (-2.4, 10.9) | 7.6 (-2.6, 18.5) | 5.6 (1.9, 9.4) | 22.1 (-0.4, 39.0) | 6.7 (3.0, 10.4) |
| Quarantine free travel | 28.1 (23.8, 32.6) | 24.5 (19.7, 29.4) | 18.9 (11.8, 26.3) | 34.1 (22.3, 47.0) | 21.4 (17.1, 25.8) | 44.8 (13.4, 68.3) | 23.1 (19.0, 27.3) |

© 2022 Fung LWY et al. *JAMA Network Open.*

**eTable 5.** Preference weights and marginal willingness to pay (mWTP) of participants stratified by place of origin.

| Attributes | Local* (n=2,381) | | Non-local (n=953) | |
|---|---|---|---|---|
| | Adjusted β** (95% CI) | mWTP*** (95% CI) | Adjusted β (95% CI) | mWTP (95% CI) |
| Cost | -0.00041 (-0.00051, -0.00032) | - | -0.00065 (-0.00080, -0.00050) | - |
| Risk of encountering a mild-to-moderate adverse event | -0.13 (-0.15, -0.11) | -39.5 (-54.4, -29.6) | -0.18 (-0.21, -0.14) | -34.9 (-47.1, -26.3) |
| Risk of encountering a severe adverse event | -0.25 (-0.29, -0.2) | -76.5 (-100.4, -59.9) | -0.24 (-0.31, -0.17) | -47.1 (-65.9, -32.7) |
| Efficacy for protection against COVID-19 infection | 0.32 (0.3, 0.34) | 99.5 (80.9, 129.2) | 0.32 (0.28, 0.35) | 61.8 (49.1, 80.1) |
| Efficacy for protection against severe manifestation from COVID-19 infection | 0.25 (0.23, 0.28) | 79.4 (64.0, 103.5) | 0.26 (0.23, 0.29) | 51.2 (41.2, 66.0) |
| Duration of protection | 0.17 (0.15, 0.18) | 51.5 (40.6, 68.2) | 0.15 (0.12, 0.17) | 29.1 (21.9, 39.1) |
| Incentive – Low value | 0.24 (0.15, 0.34) | 76.0 (47.7, 111.8) | 0.24 (0.089, 0.39) | 46.7 (17.7, 78.8) |
| Incentive – High value | 0.22 (0.077, 0.36) | 67.8 (23.7, 117.1) | 0.29 (0.085, 0.5) | 57.7 (15.5, 101.8) |
| Incentive – Quarantine free travel | 0.75 (0.59, 0.92) | 234.5 (171.8, 320.6) | 0.93 (0.69, 1.2) | 182.9 (129.5, 252.6) |
| Model specifications | Local: Log likelihood = -8,554; McFadden Pseudo $R^2$ = 0.2611 | | | |
| | Non-local: Log likelihood = -3,743.2; McFadden Pseudo $R^2$ = 0.2480 | | | |

*Local denotes Hong Kong, non-local denotes all other places of origin.

* Adjusted by age, sex, vaccination status of family or friends, COVID-19 infection status of people around participants, adverse event status after vaccination of people around participants, and block.

**Reported in USD, 1 USD = 7.8 HKD

© 2022 Fung LWY et al. *JAMA Network Open.*

**eTable 6**. Preference weights estimated by mixed logit regression model with efficacy of protection against COVID-19 infection as a categorical variable

| Attribute levels | Adjusted β* |
|---|---|
| 30% (Reference) | - |
| 50% | 0.31 |
| 75% | 0.97 |
| 95% | 1.71 |
| Model specifications | Log likelihood = -12,578; McFadden Pseudo $R^2$= 0.2402 |

**Adjusted for age, sex, vaccination status of family or friends, COVID-19 infection status of people around participants, adverse event status after vaccination of people around participants, and block.

© 2022 Fung LWY et al. *JAMA Network Open.*

**eTable 7.** Preference weights estimated by mixed logit regression model with efficacy of protection against severe manifestation of COVID-19 infection as a categorical variable

| Attribute levels | Adjusted β* |
|---|---|
| 20% (Reference) | - |
| 50% | 0.41 |
| 80% | 1.17 |
| 100% | 1.79 |
| Model specifications | Log likelihood = -12,448; McFadden Pseudo $R^2$= 0.2481 |

**Adjusted for age, sex, vaccination status of family or friends, COVID-19 infection status of people around participants, adverse event status after vaccination of people around participants, and block.

© 2022 Fung LWY et al. *JAMA Network Open.*
